# A-Mode Ultrasound-Based Prediction of Transfemoral Amputee Prosthesis Walking Kinematics via an Artificial Neural Network

**DOI:** 10.1109/TNSRE.2023.3248647

**Published:** 2023-03-08

**Authors:** Joel Mendez, Rosemarie Murray, Lukas Gabert, Nicholas P. Fey, Honghai Liu, Tommaso Lenzi

**Affiliations:** Utah Robotics Center, Department of Mechanical Engineering, The University of Utah, Salt Lake City, UT 84112 USA; Utah Robotics Center, Department of Mechanical Engineering, The University of Utah, Salt Lake City, UT 84112 USA; Utah Robotics Center, Department of Mechanical Engineering, The University of Utah, Salt Lake City, UT 84112 USA; Walker Department of Mechanical Engineering, The University of Texas at Austin, Austin, TX 78712 USA; State Key Laboratory of Robotics and Systems, Harbin Institute of Technology, Shenzhen 518055, China; School of Computing, University of Portsmouth, PO1 3HE Portsmouth, U.K.; Utah Robotics Center, Department of Mechanical Engineering, The University of Utah, Salt Lake City, UT 84112 USA

**Keywords:** A-Mode ultrasound, intent recognition, joint kinematics prediction, lower-limb prosthesis, transfemoral amputation

## Abstract

Lower-limb powered prostheses can provide users with volitional control of ambulation. To accomplish this goal, they require a sensing modality that reliably interprets user intention to move. Surface electromyography (EMG) has been previously proposed to measure muscle excitation and provide volitional control to upper- and lower-limb powered prosthesis users. Unfortunately, EMG suffers from a low signal to noise ratio and crosstalk between neighboring muscles, often limiting the performance of EMG-based controllers. Ultrasound has been shown to have better resolution and specificity than surface EMG. However, this technology has yet to be integrated into lower-limb prostheses. Here we show that A-mode ultrasound sensing can reliably predict the prosthesis walking kinematics of individuals with a transfemoral amputation. Ultrasound features from the residual limb of 9 transfemoral amputee subjects were recorded with A-mode ultrasound during walking with their passive prosthesis. The ultrasound features were mapped to joint kinematics through a regression neural network. Testing of the trained model against untrained kinematics show accurate predictions of knee position, knee velocity, ankle position, and ankle velocity, with a normalized RMSE of 9.0 ± 3.1%, 7.3 ± 1.6%, 8.3 ± 2.3%, and 10.0 ± 2.5% respectively. This ultrasound-based prediction suggests that A-mode ultrasound is a viable sensing technology for recognizing user intent. This study is the first necessary step towards implementation of volitional prosthesis controller based on A-mode ultrasound for individuals with transfemoral amputation.

## I. Introduction

TYPICAL lower-limb prostheses are passive devices that fail to fully replicate the biomechanical functions of the missing biological limb [1], [2]. Powered prostheses can potentially address the limitations of passive prosthesis by using their embedded actuators, sensors, and control system. With these powered prostheses, amputees are able to perform activities such as crossing obstacles, squatting, or walking up stairs that are not possible with passive prostheses [3], [4]. Furthermore, activities that are possible with passive prostheses, such as walking, ascending ramps, sitting down, and standing up, can become more efficient with powered prostheses [5], [6], [7], [8]. To obtain stable and efficient ambulation, powered prostheses need controllers capable of coordinating the actions of the prosthesis with the movement intention of the user.

Most powered prosthesis controllers are designed to react to the user’s movement. The motion of the robotic leg is typically guided by a finite-state machine that classifies the user’s movement intention. Basically, when the user meets certain prosthesis loading and orientation conditions the powered prosthesis switches to a different action, coherent with the interpretation of the user’s intention to move. Although most controllers rely solely on mechanical sensors [9], [10], camera systems have also been developed in an effort to improve the reliability of the user’s intention detection [11], [12], [13], [14]. Unfortunately, these techniques require training on large datasets, which can be resource intensive and scale poorly because subject specific datasets are typically necessary to improve accuracy of the classification [15], although real-time adaptation models may in part address this issue [10]. These classifiers are typically used to identify the intended ambulation mode and environment. However, they do not capture how the user wants the prosthesis to move within the identified ambulation mode.

Several control strategies have been developed to define the action of the prosthesis for specific ambulation tasks, such as walking or stair climbing. Some controllers split the gait cycle into a finite number of sequential phases and change the impedance or the position of the powered prosthesis joints accordingly between these discrete phase [16], [17], [18]. Other controllers define the gait cycle using a continuous phase evolution, enabling the users to gain some control of the powered prosthesis motion as needed, for example, when they want to change walking speed [19], [20]. Moreover, some controllers can continuously adapt the action of the powered prosthesis based on the motion of the user’s residual limb to provide indirect volitional control over walking [21] and stair ambulation [22], as needed to adapt to changing environment constraints like different step heights or different obstacle sizes. These controllers provide the users with some agency over the powered prosthesis. However, they fail to give them direct control over the prosthesis action. For example, users cannot voluntarily control the movements of the powered prosthesis when it is off the ground or voluntarily control the torque generated by the prosthesis when it is contact with the ground.

Controllers based on electromyography (EMG) can provide a direct link between the user’s muscle excitation and the action of the powered prosthesis. Surface EMGs have been used to improve the accuracy of the classifiers that determine the ambulation task intended by the user [23], [24], [25]. However, this classification strategy does not provide the user with volitional control of the prosthesis. More direct approaches, in which the EMG signals are explicitly related to the control of the prosthesis have been successfully implemented and provided users with non-weight-bearing volitional control [26], [27]. More recently, an EMG controller has been used to provide volitional control of the knee extension torque provided by a powered prosthesis enabling squatting, lunging, and sit to stand transition under different loading conditions [28], [29], [30], [31]. Despite these promising results, voluntary EMG controllers suffer from limitations related to the low signal-to-noise ratio and the lack of muscle specificity typical of surface EMG sensors [32]. A new sensing modality able to overcome these limitations might open the door to new methods for low-level direct volitional control of lower-limb prostheses.

Ultrasound sensing can address some of the limitations of EMG by providing a depth dimension and improving muscle specificity. Ultrasound transducers emit an ultrasound into the user’s limb. As the ultrasound is transmitted and travels deeper into the limb, part of the ultrasound is reflected to the transducer at the boundaries between different tissues. The amplitude of the reflection depends on the types of tissues at the boundary. Boundaries with high echogenicity, such as muscle fascia, are reflected as high-intensity values, while areas with low echogenicity, such as subcutaneous fat and muscle tissue, correspond to low-intensity values. Monitoring these boundaries during dynamic muscle contraction can be used to interpret the intention of the user. Previous work suggests that ultrasound can perform better than conventional surface EMG in instances of gesture recognition [33], discrete force estimation [33], and ambulation mode classification [34]. Additionally, ultrasound appears to be more robust against force variations [33] and muscle fatigue [35]. Two types of ultrasound technologies have been used in the application of wearable devices: B-mode ultrasound and A-mode ultrasound. The widely used B-mode ultrasound creates 2D images that provide a cross-sectional view of the user’s musculature. Image features such as muscle thickness, pennation angle, and fascicle length have been related to joint kinematics and kinetics [36], [37]. Gesture recognition via B-mode ultrasound has been successfully integrated in the control of upper-limb powered prostheses [38]. In lower-limb applications with able-bodied subjects, B-mode ultrasound sensing has allowed for the continuous classification of ambulation modes [34] and prediction of joint kinematics [39] and kinetics [40]. Furthermore, muscle fatigue [41] and muscle force [42] measurements from B-mode ultrasound have been used to determine exoskeleton assistance. Despite these promising results, the high cost and large size of B-mode transducers limit their feasibility for real-life use.

In this study, we propose using A-mode ultrasound sensing to predict the prosthesis kinematics of transfemoral amputees during level-ground walking. A-mode ultrasound is a more affordable and portable alternative to B-mode ultrasound. Thus, it can be more easily integrated into a prosthetic socket. A mode ultrasound returns a 1D array corresponding to depth. Although image features such as pennation angle and fascicle length are no longer available, echogenicity can still be interpreted through the intensity of the 1D signal [43]. This reduction in the feature space could also simplify training and allow for faster online predictions necessary for the control of powered prostheses. Multiple able-bodied studies have applied the technology to gesture recognition [44], force estimation [45], and wrist/hand kinematics estimation [46]. Furthermore, A-mode has allowed for finger gesture recognition and wrist rotation estimation with transradial amputee subjects [47], as well as ambulation mode recognition in above-knee amputee subjects [48]. However, it is not known whether ultrasound can be used to predict prosthesis kinematics in above-knee amputees. We hypothesize that A-mode ultrasound can track the muscular morphological deformations in the user’s residual limb, which can be related to the user’s walking kinematics offline. By providing the first demonstration of kinematic predictions based on A-mode ultrasound in transfemoral amputees, this study is a critical first step toward the development of powered prosthesis controllers that can give users direct voluntary control of their prostheses.

## II. Materials And Methods

### A. A-Mode Ultrasound System

For this study, we used a wearable 4-channel A-mode ultrasound system [43]. The device runs at 80 Hz with sequential readings from the 4 channels. Every 12.5 ms the ultrasound reading from one channel is updated. Thus, it takes 50 ms to receive updated information from all four channels. Each sensor has a penetration depth of 3.94 cm through soft tissue [43]. Further specifications of the device can be found in previous upper-limb studies [43].

A custom 3D printed case was created to strap the ultrasound system around the subject’s waist, with the board positioned on the ipsilateral side. The sensors were placed in custom TPU 3D printed sensor holders that were shaped to reduce loss of suction and improve user comfort.

The A-mode ultrasound system was powered by a 3-cell lithium-ion battery. The combined weight of the system, battery, and case is 440 g. The height and width of the custom case are 13 cm and 11.5 cm respectively, resulting in a surface area of 149.5 cm^2^. The case is only 4 cm thick and allows for comfortable arm swing even when placed on the side of the user’s waist.

### B. Experimental Setup

Nine transfemoral amputees were recruited for the study. The group consisted of 7 male subjects and 2 female subjects whose ages ranged from 29-74. Detailed subject information can be found in Table I.

**Fig. 1.**
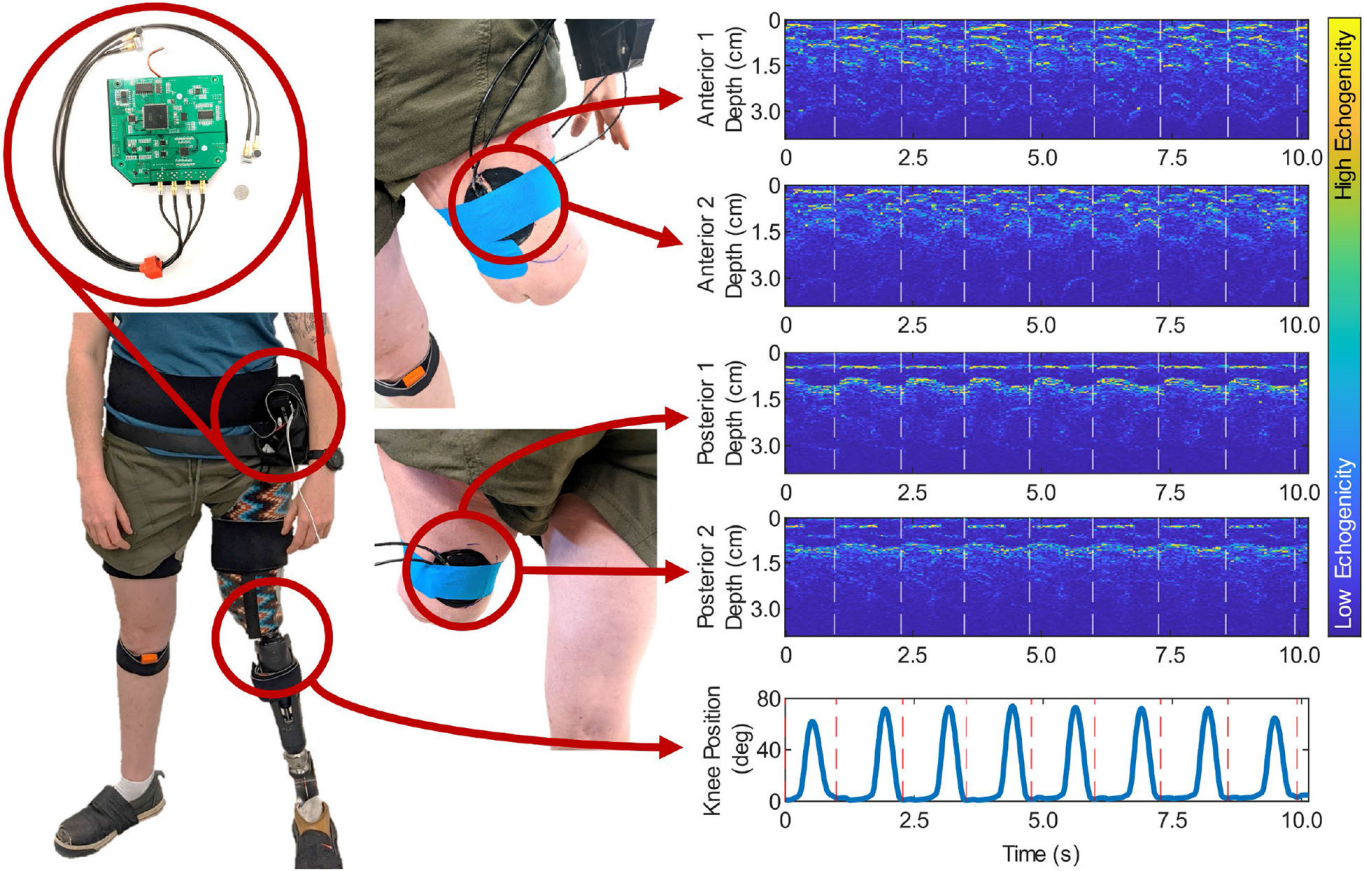
Sensor placement and A-mode ultrasound readings. The A-mode ultrasound system was secured around the subject’s waist using Velcro straps. The four A-mode ultrasound sensors were placed on the user’s residual limb. Two were placed anteriorly, and two were placed posteriorly. Subjects also donned the Xsens IMU-based motion capture system which recorded joint kinematics during walking. Representative A-mode ultrasound data and joint kinematics are demonstrated for one trial of walking, where the dashed lines correspond to the start of a new stride at heel strike.

Placement of the ultrasound sensors occurred while the subject was seated without their socket and passive prosthesis. Two sensors (channels 1 and 3) were placed anteriorly on the residual limb to target the quadricep muscle group. The other two sensors (channels 2 and 4) were placed on the posterior side to target the hamstring muscle group. The selected pairing of the channels resulted in each muscle group being sampled at 40 Hz. Specific muscles within each group were not targeted due to differences in residual limb shape and user comfort. We determined the location by first positioning the sensor on the muscle belly of the target muscle (Fig. 1). We then displaced the sensor until the 1D ultrasound signal displayed defined peaks that changed in response to the subject contracting and relaxing their muscle (Fig. 1). After a suitable location was determined, the sensors and sensor holders were secured with kinesiology tape. The subjects then rolled on their liner and donned their socket on top of the sensors. After the subjects donned their prescribed prosthesis, we asked them to stand and walk to ensure comfort. If the subject reported discomfort, we repeated the sensor placement process until we obtained a comfortable sensor placement that resulted in similar signal quality. Sample ultrasound recordings and sensor placements are depicted in Fig. 1.

**Fig. 2.**
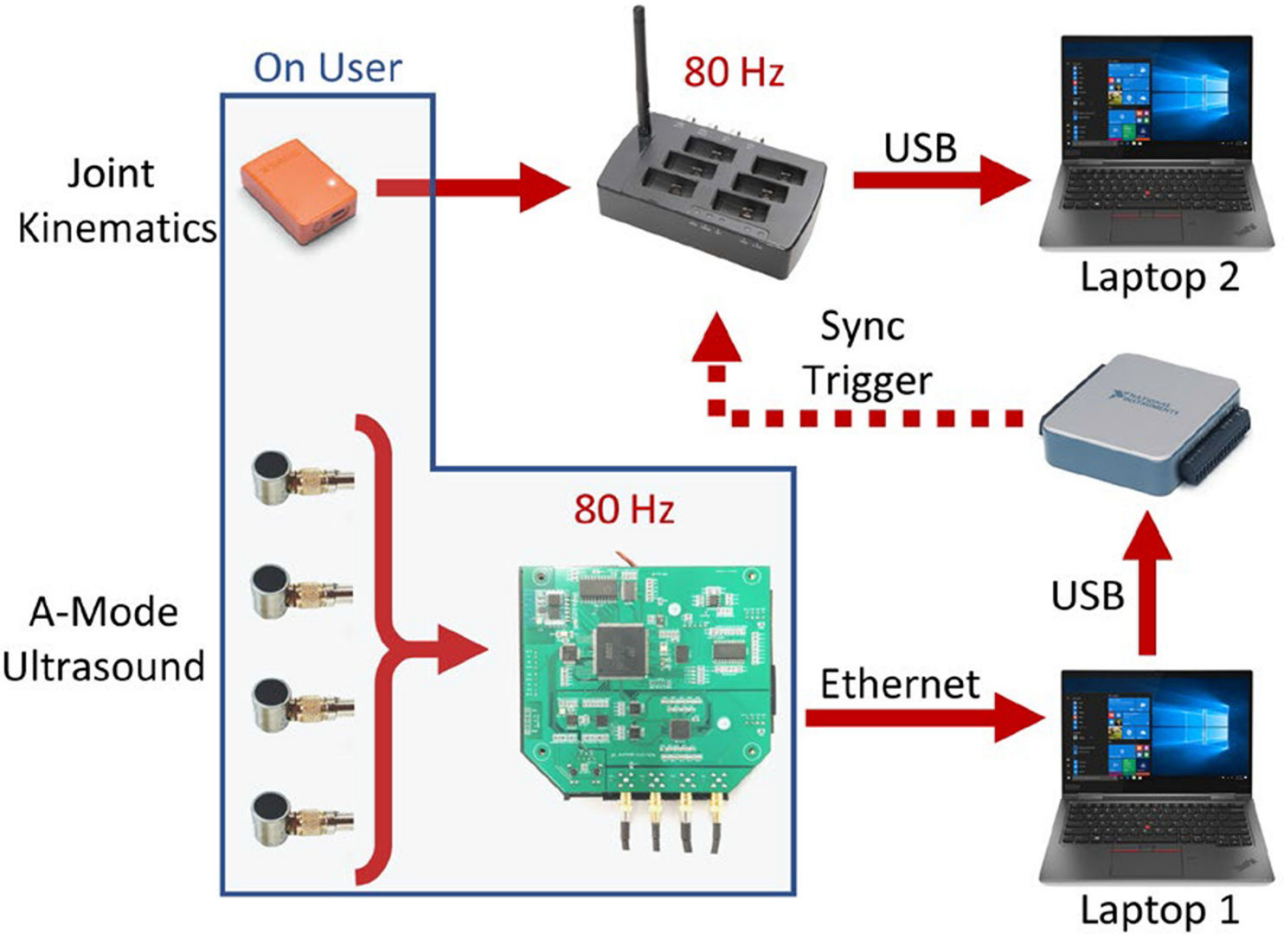
Experimental setup. Subjects donned the Xsens IMU-based motion capture system and the A-Mode ultrasound systems, whose channels were placed on the user’s residual limb. Laptop 1 received ultrasound data from the ultrasound system via an ethernet connection. Laptop 2 received the data pertaining to the user’s walking kinematics. The recordings from the two systems, both running at 80 Hz, were synchronized via a DAQ system, which received an input signal from Laptop 1 at the start of recording to trigger the recording from Laptop 2.

Once the ultrasound sensor placement was determined, the users donned an IMU-based motion capture system (Xsens MVN, Enschede, Netherlands) and performed the system calibration [49]. To match the sampling frequency of the ultrasound system, we set the sampling frequency of the motion capture system to 80 Hz. The A-mode ultrasound system transferred ultrasound data to a laptop via an ethernet cable (Fig. 2), which was long enough to allow the user to walk without any obstruction. The laptop triggered the recording of a separate laptop running the IMU-based motion capture system Xsens via a DAQ system (National Instruments USB-6001). This system ensured that the ultrasound and motion capture data were synchronized. The full experimental setup is depicted in Fig. 2.

Once the experimental setup was complete, subjects performed the 10 m walk test [50] four times. The first three tests were performed at a comfortable self-selected speed, while the last test was performed at a faster self-selected speed. The study was conducted in accordance with the Declaration of Helsinki and approved by the Institutional Review Board of The University of Utah (Protocol #00103197, approved 06/16/2021). Informed consent was obtained from all subjects involved in the study.

### C. Data Processing

**Fig. 3.**
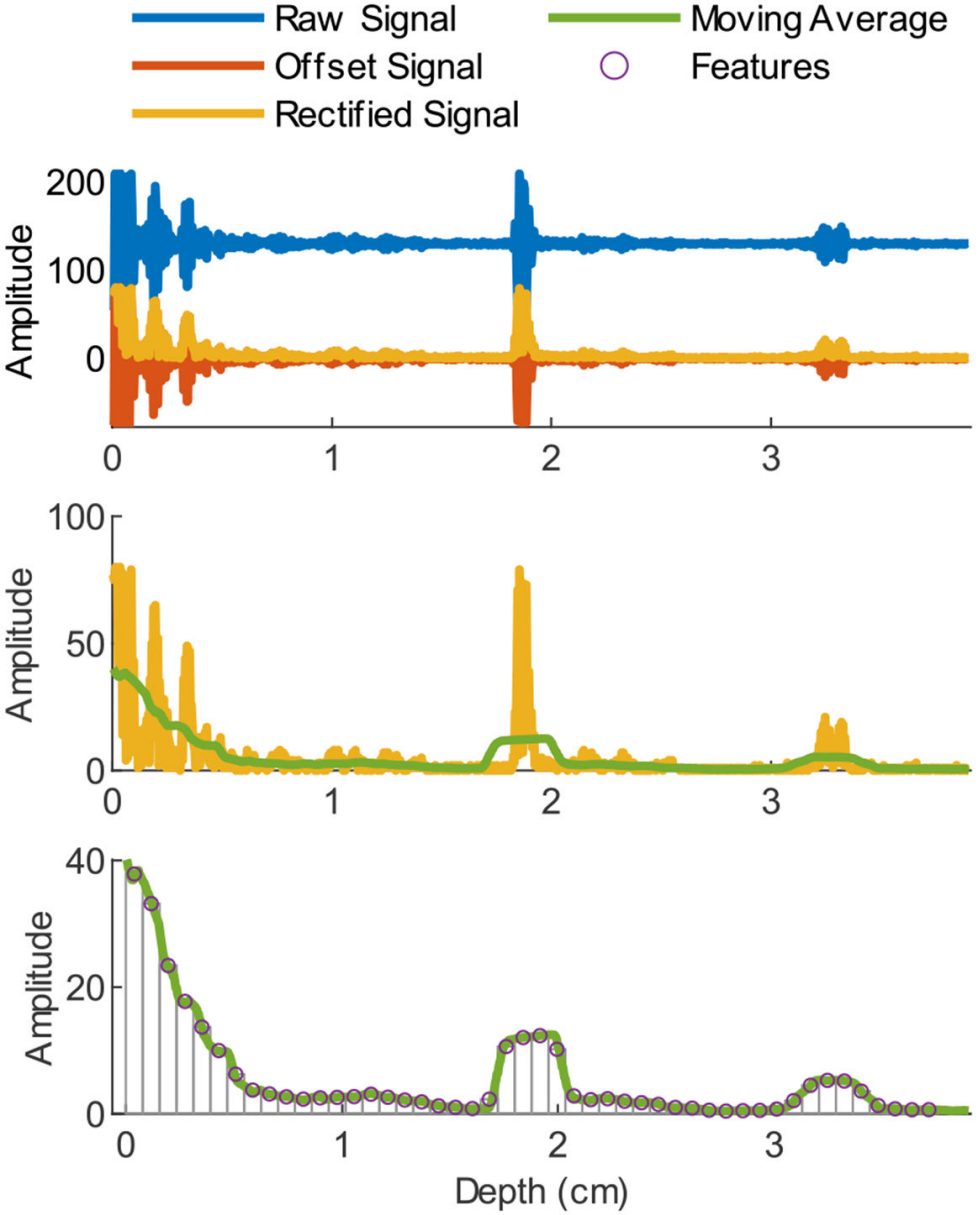
Feature Reduction. The raw ultrasound signal (blue) from each A-mode ultrasound sensor was offset (red) and rectified (yellow). A mean convolution was then applied to the rectified signal to find the envelope of the signal (green). The envelope was then segmented into 48 windows, where the average of the data points served as the feature (purple) from that window. This resulted in a total of 192 ultrasound features.

We processed the A-mode ultrasound data and the joint kinematics offline in MATLAB (Mathworks, Natick, MA, USA) in preparation for our machine learning models. The A-mode ultrasound system outputted a 1D ultrasound signal of 997 sample points iteratively throughout four channels. For each time frame, we rectified the 1D signal and applied a moving average convolution (sliding window of 77 sample points) to obtain the envelope of the signal. Next, we removed 37 sample points from the deeper end of the signal to reduce the signal to 960 sample points. We then segmented the signal into 48 windows of 20 sample points, with the mean of each window (average of the 20 sample points) serving as that window’s feature. For each time step, this process results in 48 features from the 997 sample points. Given that the 997 sample points correspond to a depth of 3.94 cm, each feature corresponds to approximately 0.08 cm. Fig. 3 depicts the full feature reduction process. The parameters used in the data processing were determined through preliminary comparisons of different feature sets and motivated by previous upper limb studies [45].

### D. Machine Learning

We created a regression neural network using MATLAB’s Statistics and Machine Learning Toolbox to find a mapping between the ultrasound features and the users’ kinematics. For each subject, we trained four individual models, corresponding to the four joint variables to be predicted: knee position, knee velocity, ankle position, and ankle velocity. In this study, the feature set comprised of the 48 most recent features from each of the four channels, for a total of 192 features. The label set included the kinematic values for the corresponding joint variable that were sampled at 80 Hz. At each sampling time using an 80 Hz clock, the ultrasound system updates 48 features of the 192 found in the feature set. As a result, each time step has a unique feature set corresponding to the kinematics of the leg. There are overlapping features within sequential feature sets due to only one channel being read at a time, but this is contained within individual 10m walking trials.

The framework of the regression neural network consisted of three 10-node fully connected layers alternated with three ReLU activation layers and a final regression output layer. This neural network structure was selected to keep computational complexity low, which facilitates translation to online prediction.

Four 10 m walking trials were recorded for each subject. All subjects performed the trials at self-selected speeds. However, the subjects were instructed to walk at their comfortable walking pace for the first three trials, and to walk at a faster pace for the fourth trial. Ultrasound data and the joint kinematics from the first two trials were used to train the regression neural networks offline. The remaining two trials served as two distinct testing sets. The testing set recorded at the normal walking speed is denoted as the normal speed testing set, while the testing set recorded at the faster speed is denoted as the fast speed testing set. No inter-subject models were trained due to residual limb and sensor placement differences. Separate models were trained for the four joint variables. Between the two testing sets, the normal speed and the fast speed testing set, the same model was used to generate our predictions. This protocol enabled us to assess how well the ultrasound-based models adapt to variations of the same activity. Training was carried out on a laptop utilizing an Intel(R) Core (TM) i7-8650U processor with a training time of 9.2 ± 2.0 s across all models. Training data varied between 652 and 1040 frames across subjects, with the difference being due to different walking speeds.

### E. Data Analysis

The performance of our predictive model was analyzed both as unfiltered and filtered data. Normalized RMSE values were calculated between the unfiltered predictions and the actual kinematics. The lack of a delay in unfiltered data provides a closer comparison with previous studies. Comparisons pertaining to the kinematic profile of the predictions and delay of the signal were calculated with filtered data. To filter the data, we applied a 2^nd^ order Butterworth filter to the A-mode ultrasound-based prediction of the joint kinematics. For this filter, we used a cutoff frequency of 7 Hz, which is within the range of optimal cutoff frequencies for walking [51]. Although a bidirectional filter could have been used for these offline filtered predictions, we chose to investigate the application of a one-way filter, as it is more representative of how the prediction could be used in an online application.

## III. Results

### A. Normalized RMSE

**Fig. 4.**
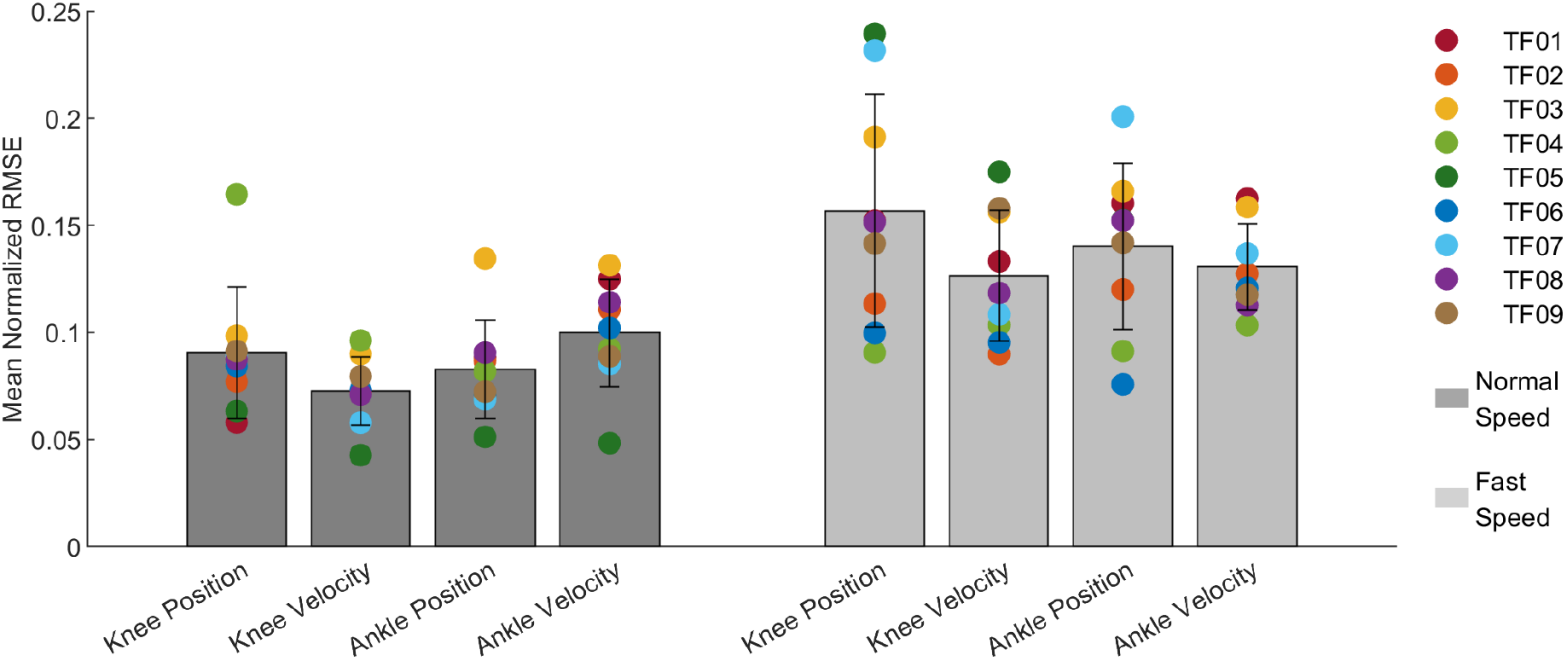
Mean Normalized RMSE of predicted kinematics. Joint kinematics were predicted for 9 transfemoral amputee subjects using two distinct testing sets: the normal speed testing set and the fast speed testing set. For the normal speed testing set, the mean normalized RMSE for the knee position, knee velocity, ankle position, and ankle velocity were 9.0 ± 3.1 %, 7.3 ± 1.6 %, 8.3 ± 2.3 %, and 10.0 ± 2.5 % respectively. For the fast speed testing set, the mean normalized RMSE for the knee position, knee velocity, ankle position, and ankle velocity were 15.7 ± 5.4 %, 12.6 ± 3.1 %, 14.0 ± 3.9 %, and 13.1 ± 2.0 % respectively. The error was calculated for the unfiltered prediction.

We performed an analysis of the four trained models by calculating the normalized RMSE of the prediction. This metric compared the unfiltered prediction to the actual kinematics. Predictions were generated for two different testing sets; the normal speed testing set and the fast speed testing set. Subjects walked with an average stride time of 1.2 ± 0.1 s during the normal speed testing set, and with an average stride time of 1.0 ± 0.2 s during the fast speed testing set. Fig. 4 shows the overall results for all four variables between the two test sets. The individual markers correspond to the mean normalized RMSE for individual subjects, while the bar graph corresponds to the mean normalized RMSE across all subjects. For the normal speed testing set, the mean normalized RMSE across all subjects for knee position, knee velocity, ankle position, and ankle velocity were 9.0 ± 3.1 %, 7.3 ± 1.6 %, 8.3 ± 2.3 %, and 10.0 ± 2.5 % respectively. This corresponds to a RMSE of 6.0 ± 2.2 deg, 60.6 ± 16.3 deg/s, 1.8 ± 0.7 deg, and 27.9 ± 11.1 deg/s. Testing the trained model on the fast speed testing set resulted in a mean normalized RMSE of 15.7 ± 5.4 %, 12.6 ± 3.1 %, 14.0 ± 3.9 %, and 13.1 ± 2.0 % for knee position, knee velocity, ankle position, and ankle velocity respectively, which corresponds to a RMSE of 11.7 ± 5.6 deg, 125.6 ± 46.6 deg/s, 3.4 ± 1.5 deg, and 46.9 ± 22.7 deg/s. The normalized RMSE for the individual subjects are detailed in Table S1, and the corresponding RMSE values are detailed in Table S2.

### B. Filtering and Delay

**Fig. 5.**
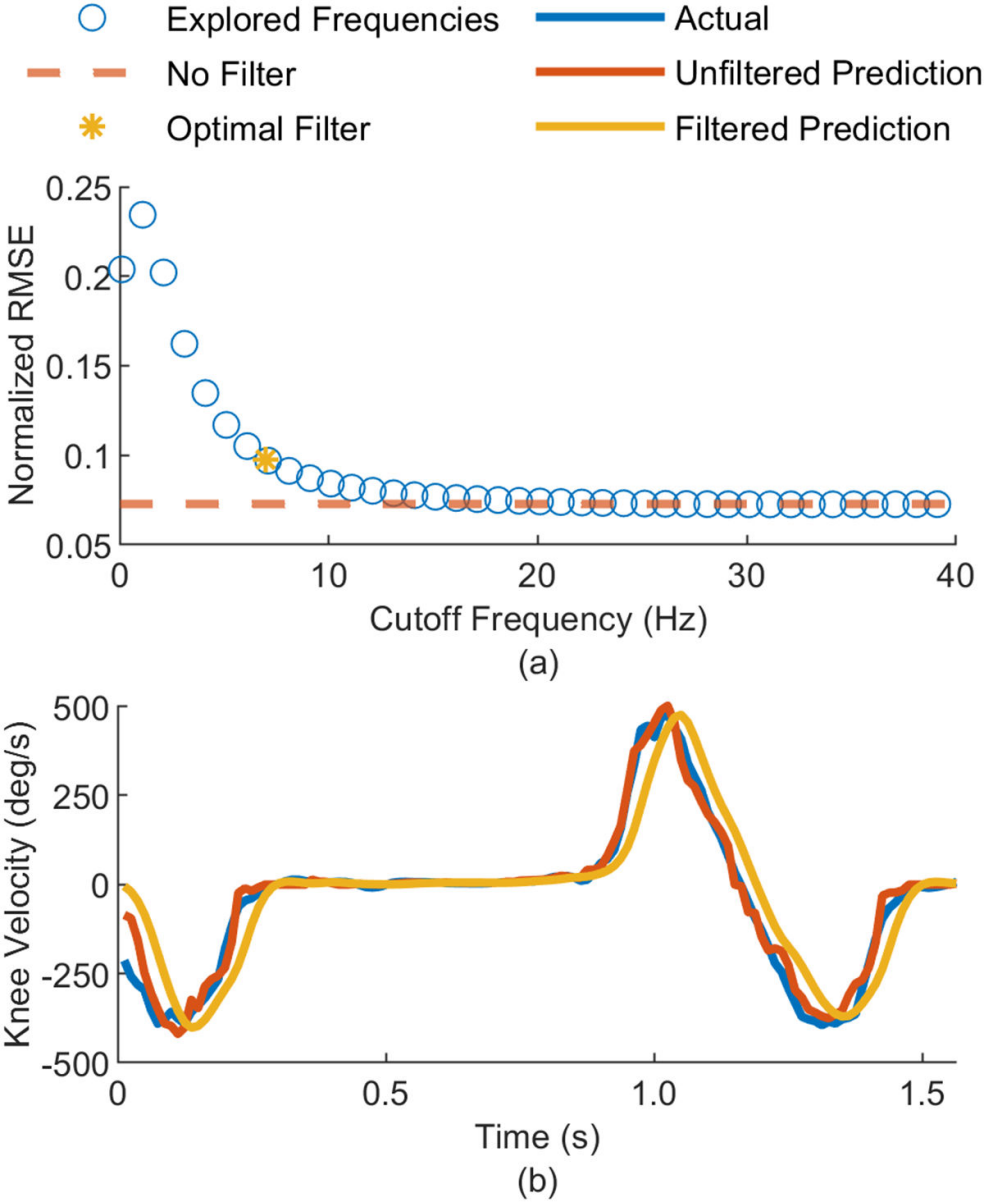
Filter Analysis. (a) Normalized RMSE for multiple cutoff frequencies (blue circles) throughout all subjects for a 2^nd^ order Butterworth filter, normalized RMSE of the unfiltered prediction (orange dashed line), and normalized RMSE while using the optimal cutoff frequency (yellow asterisk) as determined by [51]. (b) Recorded knee velocity (blue trajectory), with the unfiltered prediction (red) trajectory), and the filtered prediction (yellow trajectory).

**Fig. 6.**
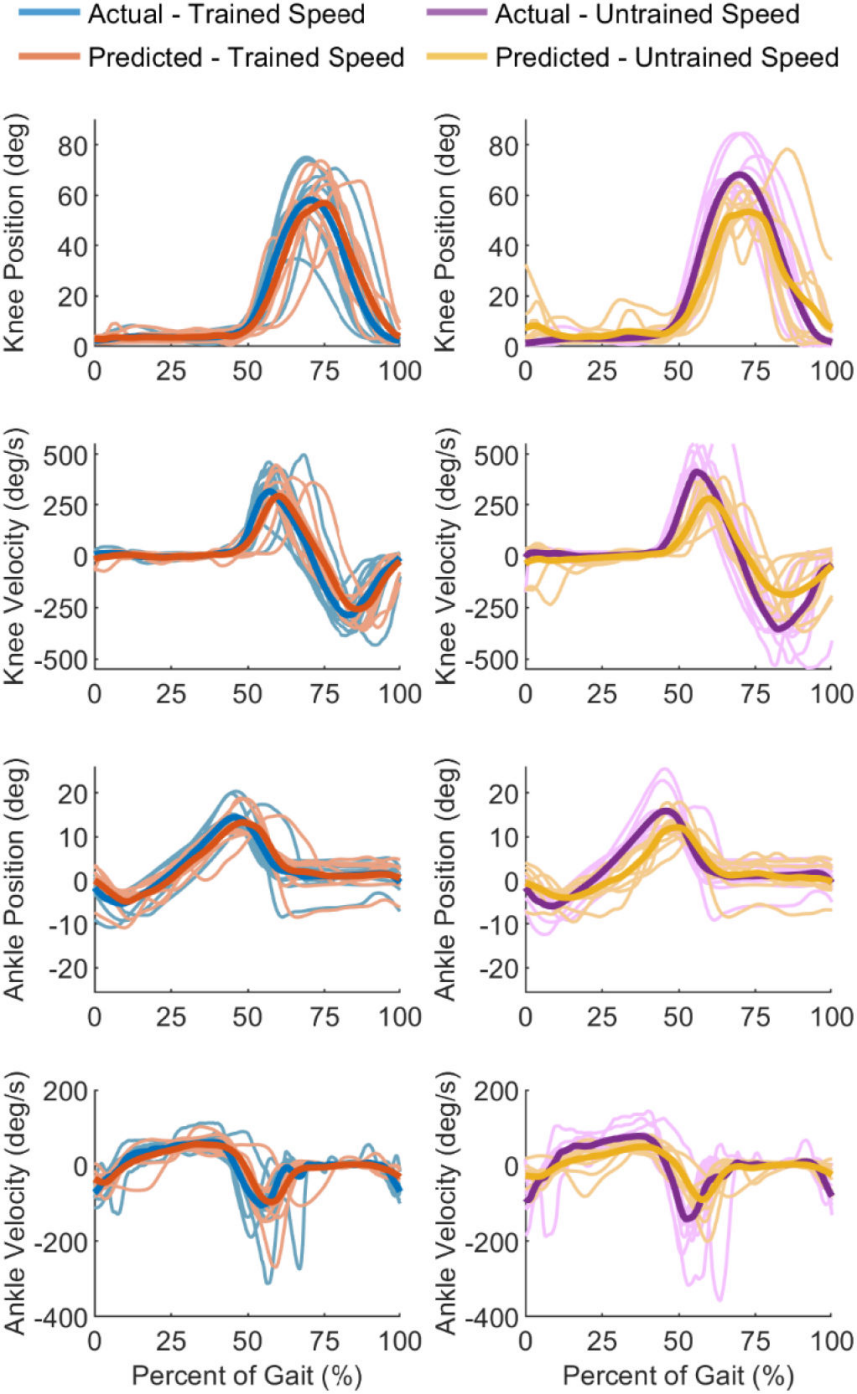
Mean recorded kinematics and mean predicted kinematics across all subjects on two distinct testing sets. The first testing set (left column) consists of data collected at a similar walking speed as that found in the training set. Both the recorded kinematics (blue) and the filtered predictions (orange) from the A-mode ultrasound data are depicted for the normal speed testing set. The second testing set (right column) corresponds to the trial whose speed was faster than that found in the training set. Both the recorded kinematics (purple) and the filtered predictions (yellow) are depicted for the fast speed testing set. Predictions are filtered using a 2^nd^ order Butterworth filter with a cutoff frequency of 7 Hz.

We analyzed the effects of different filters on the prediction error and smoothness. As shown in Fig. 5, filtering the prediction with an optimal cutoff frequency for walking resulted in a higher normalized RMSE compared to not filtering, due to the delay introduced by the filter. However, applying a filter resulted in a smoother prediction, which would be preferable for online control of powered prostheses, where users need minimal chattering and vibrations. On average across all subjects and joint variable predictions, we found a delay of 35.1 ± 5.8 ms when comparing the filtered predictions to the actual prosthesis kinematics. This delay can be seen between the actual and predicted values in Fig. 6. In the normal speed testing set, the recorded maximum dorsiflexion and maximum plantarflexion occur at 5.5% and 46.3% of gait, and the predicted maximum dorsiflexion and maximum plantarflexion occur at 8.9% and 49.5% of gait. Given that the average recorded stride time was 1.2 ± 0.1 s, this difference corresponds to 41 ms and 38 ms. The peaks of the joint velocities experience a similar delay, where the predicted maximum dorsiflexion velocity, knee extension velocity, and knee flexion velocity lag 38 ms, 38 ms, and 35 ms behind the actual peak velocities. Notably, the peaks corresponding to the maximum plantarflexion velocity and the maximum knee position do not reflect a similar delay. The recorded max knee flexion occurs at 70.7% of gait, while the predicted max knee flexion occurs at 75.0%, corresponding to a 51 ms difference. Meanwhile, the predicted maximum plantarflexion velocity occurs 6 ms earlier than the recorded maximum plantarflexion velocity. We only consider the normal speed testing set in the analysis of the delay, as increased error in the fast speed testing set predictions led to unreliable cross-correlation values.

### C. Kinematics

We compared the kinematics resulting from the filtered predictions of both testing sets. The mean joint kinematics predictions across all subjects derived from the A-mode ultrasound signals are shown in Fig. 6 for both testing sets. All four joint kinematics were predicted separately with their individual models. As a result, the predicted velocities did not match the derivative of the predicted positions.

For the testing set containing the normal speed, the filtered kinematic prediction visually matched the recorded kinematics, although a reduction in range is visible for all four joint variables. On average, the predicted peak knee flexion was 2.0% less than the recorded peak knee flexion. The average predicted knee velocity range was 91.3% of the average recorded knee velocity range. Looking at the ankle position, the predicted peak plantarflexion and the predicted peak dorsiflexion were 8.4% and 8.8% less than the actual peak values. The range of the predicted ankle velocity was 92.0% of the actual ankle velocity range.

For the fast speed testing set, the knee was predicted as only reaching 78.3% of the actual peak knee flexion. Similarly, the range of the predicted knee velocity was only 60.8% of the recorded range. The ankle plantarflexion prediction reached 76.3% of the actual peak ankle plantarflexion, while the ankle dorsiflexion prediction reached 67.5% of the recorded peak dorsiflexion. At this faster walking speed, the prediction for the ankle velocity resulted in a range that was 63.5% of the actual ankle velocity range.

## IV. Discussion

The goal of this study was to test the hypothesis that A-mode ultrasound sensing can predict the kinematics of transfemoral amputees during level-ground walking. Tests with nine individuals with a transfemoral amputation walking at their self-selected speed show that, for all joint variables, the predicted kinematics closely match the recorded walking kinematics, and follow their general shape, with a normalized RMSE between 7.3% and 10.0%. However, there is a visible discrepancy between the actual and predicted joint kinematics at the extremes of the joint values. Specifically, the prediction consistently undershoots the actual values, resulting in the total range of the predicted kinematics being between 2.0% and 8.7% smaller than the actual kinematics. Future work should address the observed reduction in range, for example, by exploring other A-mode ultrasound features or adding a gain to the model estimates.

Despite the reduced range, the prediction accuracy shown in this study with the unfiltered predictions is comparable to that of previous studies using EMG-based and B-mode ultrasound-based strategies for the prediction of joint kinematics on healthy individuals [39]. Able-bodied walking joint kinematic predictions using only EMG has been achieved with a normalized RMSE of 11.7%, 16.1%, 16.4%, and 18.2 % for knee position, knee velocity, ankle position, and ankle velocity respectively [39]. In contrast, the normalized RMSE for this study ranged from 7.3% to 10.0%. Able-bodied studies tracking eight or more muscle groups with EMG have achieved lower error values for ankle and knee position, with normalized RMSE values ranging between 2-5% [52], [53]. However, this approach is not feasible in amputees because some the muscle groups used in these studies [52], [52]are not available in amputees. Our joint predictions based on A-mode ultrasound were obtained by just targeting two muscle groups in the residual limb, above the amputation level. By reducing the number of muscle groups needed for an accurate joint prediction, A-mode ultrasound may provide an effective solution for above-knee amputee applications.

B-mode ultrasound has been used in able-bodied subjects to predict kinematics. A previous B-mode study has reported normalized RMSE of 9.1%, 11.4%, 10.8%, and 16.6% for knee position, knee velocity, ankle position, and ankle velocity, respectively [39]. Other B-mode studies have shown prediction of ankle dorsiflexion with an RMSE of 5.41 ± 1.83 deg [54] and knee position with an RMSE of 7.39 ± 2.91 deg [55]. Our A-mode study shows RMSE of 9.0%, 7.3%, 8.3% and 10.0% for knee position, knee velocity, ankle position, and ankle velocity (unfiltered predictions). Previous B-mode studies were conducted on healthy nonamputee subjects walking on a treadmill, while this A-mode study was conducted with transfemoral amputees continuously walking on level ground. Therefore, the error rates of our A-mode study are comparable to previous B-mode studies but cannot be directly compared due to the different subject pool and experimental protocol.

Although A-mode ultrasound does not capture the 2-D cross-sectional features as B-mode does, this study suggests that a regression neural network trained solely on the 1-D A-mode ultrasound data can accurately predict the walking kinematics of transfemoral amputees. The fact that this was accomplished via signals from the residual limb is significant, in that A-mode ultrasound can successfully sense the morphological deformation of the residual limb muscles. These deformations can be due to voluntary activation or excitation of the user’s muscle, or due to different loading conditions on the prosthesis that would, in turn, lead to various interaction forces from the user’s socket. A-mode ultrasound has a greater potential than B-mode ultrasound to be used for controlling powered prostheses due to its smaller size and portability. Future studies should aim to integrate A-mode probes into the user’s socket using similar techniques to EMG sensors.

We tested the trained regression neural net with an additional testing set to see how well the model generalized to different walking speeds. Similar to the normal speed testing set, the fast speed testing set shows a reduction in the range of its joint variable predictions (Fig. 6). However, the reduced range of motion is more pronounced in the fast speed testing than the normal speed testing set (Fig. 6). This result is likely due to the larger ranges in the recorded joint kinematics that commonly result from walking at a faster speed with a prosthesis. Thus, it is possible that the large deviations from the actual trajectories are due to extrapolating to ultrasound features that were only generated when walking at higher speeds. Because these features are unfamiliar to the model, it fails to predict the proper kinematics for walking at a faster speed. Thus, although the prediction at a higher speed captures the general shape of the recorded trajectory, the inclusion of variable speeds in the training set may be needed to improve model generalization.

The measured delay between the recorded and filtered predictions was 35 ± 5 ms. Variation in the measured delay exists due to the variation in the prediction accuracy across subjects and variables. These differences in the delays can be attributed to the slight inaccuracies in the shape of the predictions. Previous lower-limb studies have shown that a delay of 90 ms did not perturb subjects and still allowed for successful task transitions [56]. Thus, the average delay of 35 ms should not inhibit the use of A-mode ultrasound for online control of powered prostheses.

The current A-mode sensor placement resulted in accurate predictions of the joint kinematics. However, a different sensor placement may yield better results. For example, all four sensors can be paired together to target the same muscle, allowing for more information from a single muscle group but less information from across the residual limb. Alternatively, all four sensors can be placed on separate muscles, reducing the individual muscle sampling while better capturing the activity across the entire residual limb. In our study, the sensor placement was affected by user’s comfort. We expect that using custom sockets that accommodate the ultrasound sensors could improve user comfort and allow for better placement of the sensors. Custom sockets could also minimize the interaction forces between the sensors and the residual limb, reducing the sensor noise.

Additional studies are necessary to measure the performance of A-mode sensing in a real-world setting. The features used in this study were determined through preliminary analysis of different feature sets, partly motivated by previous upper limb studies. However, optimizing the machine learning framework, either by exploring different feature sets or algorithms, could improve prediction accuracy. Fatigue is another factor that may affect signal fidelity and was not considered in this study. Furthermore, the methodology presented in this study should be applied to other activities to test its viability in a real-world environment. These additional studies are necessary to understand the capabilities of A-mode ultrasound sensing in recognizing user intent.

Finally, the results presented in this study suggests that A-mode ultrasound sensing could be used to recognize the user’s motion intent. Such intent recognition is an important first step in introducing the user’s volition to the low-level control of lower limb powered prostheses. Previous studies involving EMG-based volitional control of a prosthetic knee have achieved a tracking error of 6.20 ± 0.71 deg, and recorded a tracking error of 5.20 ± 1.00 deg for the intact side [26]. The study presented in this paper resulted in a knee position RMSE of 6.2 ± 2.2 deg. Despite being an offline prediction, this result suggests that A-mode ultrasound could be used to generate control signals that capture the user’s volition. To further validate this claim, online studies involving control of an actual lower-limb powered device through A-mode ultrasound sensing should be conducted. Future studies should consider controlling the joint kinematics both directly and indirectly, using a shared control framework [26], [27], [28], [29], [30], [31].

## V. Conclusion

Direct volitional control of powered prostheses has the potential to improve the agency and mobility of users. To achieve this goal, we need a control system that can interpret the user’s intention to move. A-mode ultrasound can serve this purpose by sensing the muscular morphological deformations in the residual limb. Ultrasound technology is an appealing solution due to its deep muscle resolution and its ability to clearly distinguish between different muscle areas. This study presents the first application of A-mode ultrasound for the prediction of lower-limb joint kinematics in transfemoral amputee subjects. A regression neural network was trained using ultrasound and joint kinematic data that were recorded during walking at freely modulated overground walking speeds. The trained models demonstrate accurate predictions of position and velocity for both the knee and ankle joint when applied to a testing set. These accurate predictions demonstrate the possibility of providing direct control of the joint kinematics to the users via ultrasound sensing. Future studies will investigate the integration of A-mode ultrasound into lower-limb powered prostheses and the development of ultrasound-based volitional controllers.

## Supplementary Material

supp1-3248647 Table S1. Normalized RMSE of joint variable predictions
Table S2. RMSE of joint variable predictions

## Figures and Tables

**TABLE I:** Subject Information

Subject	Age (years)	Weight (kg)	Height (m)	Sex	Socket
TF01	29	65	1.8	Male	Suction
TF02	74	80	1.7	Male	Osseointegration
TF03	45	95	1.9	Male	Suction
TF04	68	70	1.7	Male	Suction
TF05	32	59	1.6	Female	Lanyard
TF06	32	77	1.8	Male	Suction
TF07	53	100	1.9	Male	Suction
TF08	54	78	1.7	Male	Suction
TF09	31	59	1.7	Female	Lanyard
