# Supplementary material for: A-Mode Ultrasound-Based Prediction of Transfemoral Amputee Prosthesis Walking Kinematics via an Artificial Neural Network
Source: IEEE Trans Neural Syst Rehabil Eng. Author manuscript; Available in PMC 2024 Aug 24. (PMC10447627; doi:10.1109/TNSRE.2023.3248647)
Supplement: supp1-3248647 — Table S1. Normalized RMSE of joint variable predictions Table S2. RMSE of joint variable predictions [file NIHMS1881068-supplement-supp1-3248647.pdf]

SUPPLEMENTARY MATERIAL

Table S1. Normalized RMSE of joint variable predictions

		NRMSE									
		TF01	TF02	TF03	TF04	TF05	TF06	TF07	TF08	TF09	Avg.
Normal Speed	Knee Position (deg)	5.8%	7.7%	9.8%	16.5%	6.3%	8.4%	8.9%	8.7%	9.1%	9.0±3.1%
	Knee Velocity (deg/s)	7.2%	7.2%	9.0%	9.6%	4.3%	7.3%	5.8%	7.1%	8.0%	7.3±1.6%
	Ankle Position (deg)	8.8%	8.7%	13.4%	8.2%	5.1%	7.1%	6.9%	9.1%	7.2%	8.3±2.3%
	Ankle Velocity (deg/s)	12.5%	11.1%	13.1%	9.2%	4.8%	10.2%	8.5%	11.4%	8.9%	10.0±2.5%
Fast Speed	Knee Position (deg)	15.2%	11.3%	19.1%	9.1%	23.9%	10.0%	23.2%	15.2%	14.2%	15.7±5.4%
	Knee Velocity (deg/s)	13.3%	9.0%	15.6%	10.3%	17.5%	9.5%	10.8%	11.8%	15.8%	12.6±3.1%
	Ankle Position (deg)	16.0%	12.0%	16.6%	9.1%	15.2%	7.6%	20.1%	15.2%	14.2%	14.0±3.9%
	Ankle Velocity (deg/s)	16.2%	12.7%	15.8%	10.3%	13.7%	12.1%	13.7%	11.3%	11.8%	13.1±2.0%

Table S2. RMSE of joint variable predictions

		RMSE									
		TF01	TF02	TF03	TF04	TF05	TF06	TF07	TF08	TF09	Avg.
Normal Speed	Knee Position (deg)	3.2	4.8	7.1	10.9	4.7	5.8	6.5	5.4	5.1	6.0±2.2
	Knee Velocity (deg/s)	55.7	52.1	88.8	77.2	37.5	64.9	41.9	68.2	59.3	60.6±16.3
	Ankle Position (deg)	2.3	1.6	3.5	1.5	1.7	1.8	1.5	1.4	1.1	1.8±0.7
	Ankle Velocity (deg/s)	41.1	21.8	51.1	27.1	21.9	26.2	16.2	25.0	20.9	27.9±11.1
Fast Speed	Knee Position (deg)	10.1	7.0	14.6	6.2	21.1	7.1	20.0	10.5	8.8	11.7±5.6
	Knee Velocity (deg/s)	124.2	68.1	199.8	89.3	193.0	90.1	91.9	137.3	136.6	125.6±46.6
	Ankle Position (deg)	4.7	2.3	4.6	1.8	5.9	2.0	4.6	2.6	2.4	3.4±1.5
	Ankle Velocity (deg/s)	70.0	24.9	90.9	31.4	64.8	34.8	34.6	30.6	39.9	46.9±22.7
